# Impact of water stress on the demographic traits and population projection of Colorado potato beetle

**DOI:** 10.3389/fphys.2023.1148129

**Published:** 2023-05-15

**Authors:** Xia Liu, Hangxin Yang, Fushuai Niu, Hanhan Sun, Chao Li

**Affiliations:** ^1^ Key Laboratory of Prevention and Control of Invasive Alien Species in Agriculture and Forestry of the North-western Desert Oasis (Co-construction by Ministry and Province), Ministry of Agriculture and Rural Affairs, College of Agronomy, Xinjiang Agricultural University, Urumqi, China; ^2^ Western Agricultural Research Center, Chinese Academy of Agricultural Sciences, Changji, China

**Keywords:** *Leptinotarsa decemlineata* (Say), water stress, oviposition selection, growth and development, reproduction

## Abstract

**Introduction:** The Colorado potato beetle is one of the famous quarantine pests in China which is extremely destructive to Solanaceae crops and causes serious losses to the potato industry.

**Methods:** In this experiment, the host plant potato was subjected to different degrees of water stress to observe the oviposition selection, growth and development, survival, reproduction and population growth of Colorado potato beetles.

**Results:** The results showed that adult Colorado potato beetles laid more eggs on potato plants suitable for water treatment, but fewer eggs on potato plants treated with water stress. The developmental duration of Colorado potato beetles in light drought treatment was shorter than that in control treatment, and the survival rate was higher than that in control treatment. With the aggravation of water stress, the developmental duration was prolonged, survival rate was decreased, and the number of eggs was decreased. Under different water stress levels, the intrinsic rate of increase (*r*), finite rate of increase (*λ*), net reproductive rate (*R*_0_), and mean generation time (*T*) of the Colorado potato beetle population were significantly lower than those of control treatment, but there was no significant difference between light drought and control treatment. The TIMING-MS Chart program was used to predict the population dynamics of Colorado potato beetle for 110 days, which showed the fastest population growth in CK treatments and the slowest in HD treatments. The reduced water content of the leaves also reduces the survival rate of adult Colorado potato beetles. The growth, development, survival, and reproduction of Colorado potato beetles are affected by water stress of host plants. Moderate and heavy droughts have negative effects on the development and reproduction of Colorado potato beetles.

**Discussion:** This information can be used to clarify the impact of water stress on the growth, development and population dynamics of Colorado potato beetle, to provide a theoretical basis for the control of this pest.

## 1 Introduction

Colorado potato beetle (*Leptinotarsa decemlineata* (Say)) is a famous quarantine pest in China. It is a world-wide pest that seriously harms solanaceous crops (Zhang et al., 2020); it originated in the United States, and in China, the quarantine pest Colorado potato beetle was first found in Yili Valley and the Tacheng area of Xinjiang in 1993 (Amanulla, 2015). Both adults and larvae feed on the blade, and when the damage is serious, the entire leaf can be eaten up, leaving only the stems of the plant, which is extremely destructive to potatoes (Alyokhin et al., 2008; Li et al., 2014), causing serious losses to the local potato industry (Li et al., 2013; Zhang et al., 2018).

Xinjiang is located in the northwest of China, a typical temperate continental climate zone. Water is one of the main problems faced by Xinjiang’s planting industry. Potato is a crop with strong suitability, high yield, and rich nutrition. With the proposal of the strategy of potato as a staple grain, potato, wheat, corn, and rice are called the four staple grains (Wang et al., 2019). Due to the unique geographical location and soil climate of Xinjiang, potato crops grown in Xinjiang have high yields. At the same time, Xinjiang is located in the core area of the “Silk Road Economic Belt” and has frequent trade exchanges with countries along the belt, which will increase the likelihood of the Colorado potato beetle spreading more widely (Liu et al., 2018; Liao et al., 2022a).

In recent years, the acceleration in global warming has had a significant impact on the natural ecosystem, and the corresponding changes of insect populations have also taken place (Cao et al., 2011). The interaction between plants and plant-feeding insects is a common phenomenon in nature; plants are the main food source and oviposition grounds for insects, and in general, the choice of the host is important for the growth and development of its offspring, The relationship between female insect selection on host plants and the survival and development of their offspring generally supports the preference–performance hypothesis (Jaenike, 1978; Clark et al., 2011). In the process of plant growth and development, plants will often suffer from some adverse stresses. Common abiotic stresses include high temperature, salt, and water. Water is a very important factor, which can independently affect each part of this interaction relationship and also through the interaction relationship between indirectly affect various parts. However, insects are more susceptible to water stress due to their small size (Addo-Bediako et al., 2001). Plants are the places for growth, development, reproduction, and habitation of insects, and the changes of their external morphology and internal organizational structure directly affect the living environment of herbivorous insects and then affect the behavior, growth, and reproduction of insects. Cabrera et al. (1995) found that the growth rate and relative growth rate of *Schizaphis graminum* Rondani decreased to a certain extent after feeding on wheat subjected to persistent water stress. Dang et al. (2009) found in the study of *Holotrichia oblita* Fald that when the environmental humidity was high, the larval mortality of *H*. *oblita* increased greatly. Water stress will also affect the fecundity of insects, and too low environmental humidity will affect the oviposition rate and emergence rate of insects. For example, the egg carrying rate and egg hatching rate of *Cnaphalocrocis medinalis* Guenee are significantly reduced under a low-humidity environment (Xu et al., 1999), and high humidity will have an adverse effect on the attachment of eggs. At low humidity, the pupal weight of *Spodoptera litura* Fabricius was lower and female adult spawn ability decreased (Zhong et al., 2001). Water stress will also lead to a decrease in the weight of *Nilaparvata lugens* Stal adults, and both egg laying and egg hatching rates also decreased (Luo et al., 2012). In addition, water stress also indirectly affected natural enemies. After potato drought treatment, the fecundity and life parameters of Colorado potato beetles were adversely affected, and the life and survival rate of adults of natural enemies of *Arma chinensis* Fallou were also reduced (Liu et al., 2022).

With the global climate change, the frequency and intensity of drought events may increase, so it is important to study the effects of plant-mediated herbivorous insect interactions under water stress. At present, although there are many studies on the impact of water stress on potatoes (Wu et al., 2022; Zhang et al., 2022), the impact of water stress on Colorado potato beetles and other pests is rarely reported. This experiment was conducted to study the oviposition preference, growth, development, and reproduction characteristics of Colorado potato beetle under different water stress conditions in the host plant potato, in order to clarify the impact of water stress on the growth, development, and population dynamics of Colorado potato beetle, to provide a theoretical basis for the control of this pest.

## 2 Materials and methods

### 2.1 Test materials

Potted potato plants were planted under the same management conditions (one plant is retained), the variety is “Holland 15,” and the experiment will be started after the plant growth is stable. Insects tested: Adult Colorado potato beetles were collected from the potato field in Jimsar County, Changji Hui Autonomous Prefecture, Xinjiang, and placed in an artificial climate box (RXM-168C-1 climate chamber, Ningbo Jiangnan Instrument Factory, Ningbo, China). The feeding was performed under the following conditions: ambient temperature 27 °C ± 1 °C, relative humidity 70% ± 5%, and photoperiod 16L:8D. The adults collected outdoors are the first generation. The eggs laid by the outdoor adults develop into second-generation adults. The second generation of Colorado potato beetle larvae was collected from the potato leaves planted in the earlier stage for feeding.

### 2.2 Experiment method

#### 2.2.1 Drought treatment of potato

The experiment adopted the potting soil cultivation method, planting potatoes in plastic pots (diameter 27 cm, bottom diameter 17 cm, and height 19 cm), three holes were punched at the bottom of the plastic pot to prevent water accumulation, each pot was filled with 4 kg of nutrient soil, and the potted seedlings were placed outdoors in a rain-sheltered place, which could cover from rain on rainy days and normal light on sunny days. On 28 March 2022, seed potato chunks with basically the same size and full buds were selected for sowing, and one plant per pot was planted. The pots were kept at a distance to avoid interference caused by the canopy of the grown potatoes. After sowing, they were watered enough to ensure normal emergence, and when the potato plants grow to about 10 cm (May 22), different degrees of water stress tests were carried out. According to the soil water content to determine the water stress intensity, the test set up a total of four treatments, normal irrigation (CK): the soil water content is more than 80%; light drought (LD): a soil moisture content of 55%–65%; moderate drought (MD): a soil moisture content of 35%–45%; and heavy drought (HD): 15%–25% soil water content. During the test, the soil moisture meter was used to determine the soil moisture content, and the soil water content was controlled by the weighing method, and the control group was irrigated every 4 days and the drought group was irrigated every 2 days, and the experiment was started after plant stress for 21 days (Jiao et al., 2011; Liu et al., 2022).

#### 2.2.2 Oviposition selection of Colorado potato beetles

When the potato plant grows to 10 cm, a canopy shall be set up, two pots of CK, light-drought, moderate-drought, and heavy-drought potato plants shall be placed in each canopy for the selection of the Colorado potato beetle adult egg laying host, and each pot of potato in the canopy shall be placed in a fixed position. The Colorado potato beetle adults were paired, and four Colorado potato beetle adults were placed (2♀ + 2♂); after they laid eggs on the potato plant, the number of egg pieces and eggs at 10 o’clock every morning for 7 consecutive days was recorded, and each treatment was repeated five times.

#### 2.2.3 Effects of water stress on the growth and development of Colorado potato beetles

The first instar larvae of Colorado potato beetle hatched on the same day from the indoor Colorado potato beetle population were collected and divided into four groups, CK, LD, MD, and HD; each group of 30 first instar larvae were transferred in a 9 cm plastic culture dish, with 10 replicates for each group, and the larvae used the potato leaves treated with different water in the early stage for feeding. When the larvae grow to the fourth instar and no longer feed, they are placed in a 500 mL insect rearing box to pupate and emerge. Sand is filled in the insect box to 3 cm of the mouth of the bowl, and a proper amount of distilled water is sprayed to ensure that the soil has a certain humidity so that it can emerge into adults (Li, 2013). The top is tied with gauze to prevent Colorado potato beetles from escaping. Potato leaves are placed in the insect box and replaced regularly every day. After the adults emerge, they are paired according to the ratio of male to female 1:1. Survival of each pest state of Colorado potato beetles and the oviposition of adults is recorded every day.

#### 2.2.4 Life parameter analysis

The population dynamic parameters are intrinsic rate of increase (*r*), finite rate of increase (*λ*), net reproductive rate (*R*_0_), and mean generation time (*T*). The calculation formula of the main population growth parameters is as follows (Chi and Liu, 1985; Liao et al., 2022b):

$$Intrinsic\;rate\;of\;increase\;\left(r\right):\sum_{x=0}^{\infty }e^{-r\left(x+1\right)}l_{x}m_{x},$$ (1)

$$Finite\;rate\;of\;increase\;\left(\lambda \right):\lambda =e^{r},$$ (2)

$$Net\;reproductive\;rate\;\left(R_{0}\right):R_{0}=\sum_{X=0}^{\infty }l_{x}m_{x},$$ (3)

$$Mean\;generation\;time\;\left(T\right):T=\frac{\ln\left(R_{0}\right)}{r}.$$ (4)

#### 2.2.5 Population projection

The population size and stage composition were projected from an initial population of 10 eggs on all four treatments using the TIMING-MS Chart program in 110 days (Chi, 2019). This computer program is available for download at http://140.120.197.173/ecology/.

### 2.3 Data analysis

The data for the Colorado potato beetles were analyzed using SPSS 26.0. The one-way ANOVA was used to analyze the test of between-subject effects of different water stresses. The data on the oviposition selection, development, and population parameters of Colorado potato beetles on different treatments were tested for normality and variance homogeneity. The LSD multiple mean comparison method was used to compare significant differences among different treatments that met the assumptions. Nonparametric tests were used for the data that did not meet the assumptions, the Kruskal–Wallis test was used, the pairwise comparison method was used to compare significant differences among different treatments, and the *p*-value is the value after Holm–Bonferroni correction. Survival data were analyzed by constructing survival curves using the Kaplan–Meier estimator, and comparison was performed using the log-rank test. The figures were drawn using GraphPad Prism 9.0.

## 3 Results and analysis

### 3.1 Effect of water stress on oviposition selection of Colorado potato beetle

**FIGURE 1:**
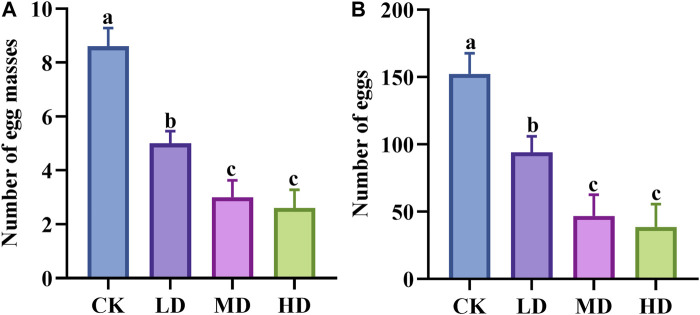
Effects of water stress on Colorado potato beetle oviposition. **(A)** Number of egg masses. **(B)** Number of eggs. CK: control group; LD: light drought; MD: moderate drought; and HD: heavy drought. After one-way ANOVA, the data were analyzed by LSD (*p*<0.05), and the letters a/b/c were used to indicate significance; data were presented as mean ± SE, *N* = 5.

There were significant differences in the egg laying capacity of Colorado potato beetles on potato plants treated with different water stresses (*p* = 0.000) (Figures 1A, B). The number of egg mass of adult Colorado potato beetles in potato plants under CK and LD treatment was significantly higher than that under MD and HD treatment. Studies on egg number and egg mass have similar results.

### 3.2 Effect of water stress on the development duration of Colorado potato beetle

**FIGURE 2:**
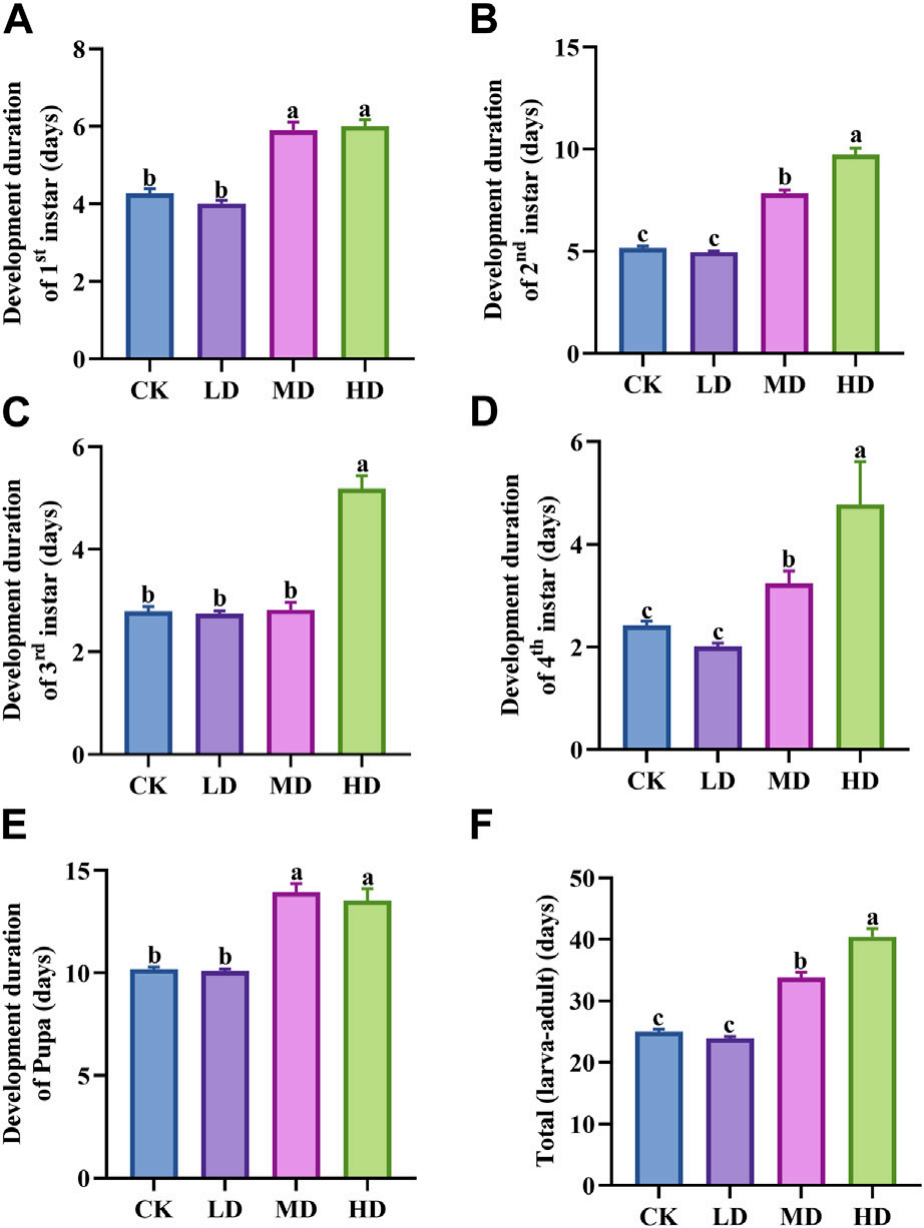
Override for the duration of Colorado potato beetle under water stress. **(A)** Development duration of first instar larva, **(B)** development duration of second instar larva, **(C)** development duration of third instar larva, **(D)** development duration of fourth instar larva, **(E)** development duration of pupa, and **(F)** total development duration refers to the sum of the development period from the first instar larva to the pupa of Colorado potato beetle. After one-way ANOVA, the data were analyzed by LSD (*p*<0.05), and the letters a/b/c were used to indicate significance; data were presented as mean ± SE, *N* = 10.

Water stress has a clear effect on the development of Colorado potato beetle (Figure 2). Under different water stress treatments, the development duration of the second instar larvae (*p* = 0.000) and the fourth instar larvae (*p* = 0.000) of Colorado potato beetles was significantly different (Figures 2B, D). Among them, the development duration of second and fourth instar larvae under MD and HD treatment was significantly longer than that of CK and LD, and there was no significant difference between CK and LD treatment. The development duration of first instar larvae (*p* = 0.000), third instar larvae (*p* = 0.000), and pupae (*p* = 0.000) was also significantly different (Figures 2A, C, E). The developmental duration of first instar larvae, third instar larvae, and pupae treated with MD and HD was significantly longer than that of CK and LD. The total development duration (*p* = 0.000) under MD and HD treatment is significantly longer than under CK and LD, and there is no significant difference between the two treatments (Figure 2F).

### 3.3 Effects of water stress on the survival rate of Colorado potato beetle

**FIGURE 3:**
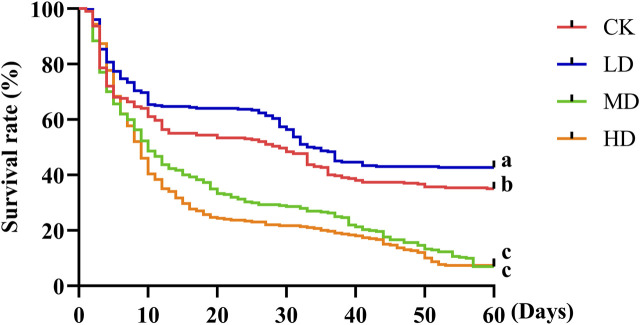
Survival rate of Colorado potato beetle under water stress. Kaplan–Meier survival curves of Colorado potato beetle fed with potato leaves that had been under water stress. The log-rank test was used to assess the significance of differences between the two survival curves.

The survival rate of Colorado potato beetle under water stress was significantly different. There were differences between CK and LD (*p* = 0.033) and MD (*p* = 0.000) and HD (*p* = 0.000) and no difference between MD and HD (Figure 3). Under water stress, the survival rate of all treatments decreased with the increase in stress time. At the 5th day of water stress, the survival rate of CK treatment and LD treatment was basically the same. After this period, the survival rate of LD treatment showed a downward trend and was always higher than that of CK treatment. After the 5th day of water stress, the survival rate of MD treatment showed a downward trend and was always higher than that of HD treatment, but on the 57th day, the survival rate of both treatments was basically unchanged and significantly lower than that of CK and LD treatment. In general, the survival rate of Colorado potato beetles decreased after water stress, but the survival rate of Colorado potato beetles on LD-treated plants was significantly higher than that of other water stress treatments after the 5th day. In conclusion, the survival rate of Colorado potato beetles decreased with the increase in water stress, and LD was beneficial to the survival of Colorado potato beetles.

### 3.4 Effect of water stress on the demographic parameters of Colorado potato beetle

There were significant differences in intrinsic rates of increase (*r*) (*p* = 0.000), finite rates of increase (*λ*) (*p* = 0.000), and net reproductive rates (*R*_0_) (*p* = 0.000) of Colorado potato beetle under different treatments. There were no significant differences in mean generation time (*T*) (*p* = 0.355) of Colorado potato beetle under different treatments (Table 1). Among them, the Colorado potato beetles under CK treatment net reproductive rates were the highest and the mean generation times was the longest, while the net reproductive rates under HD treatment were the lowest and the mean generation times were the shortest.

**TABLE 1:** Population parameters of Colorado potato beetle under water stress.

Water stress	(Intrinsic rate of increase) *r*	(Finite rate of increase) *λ*	(Net reproductive rate) *R*_0_	(Mean generation time) *T*
CK	0.0436 ± 0.01 a	1.0449 ± 0.01 a	14.6300 ± 4.09 a	48.9156 ± 0.68 a
LD	0.0234 ± 0.01 a	1.0238 ± 0.01 a	3.3467 ± 0.72 a	44.4931 ± 2.49 a
MD	0.0058 ± 0.00 ab	1.0059 ± 0.00 ab	0.9367 ± 0.33 ab	25.5648 ± 8.55 a
HD	0.0011 ± 0.00 b	1.0011 ± 0.00 b	0.5567 ± 0.19 b	25.0868 ± 8.39 a

Data were presented as mean ± SE; different lowercase letters in the same column indicate significant difference (*p* < 0.05).

### 3.5 Effect of water stress on the population projection of Colorado potato beetle

**FIGURE 4:**
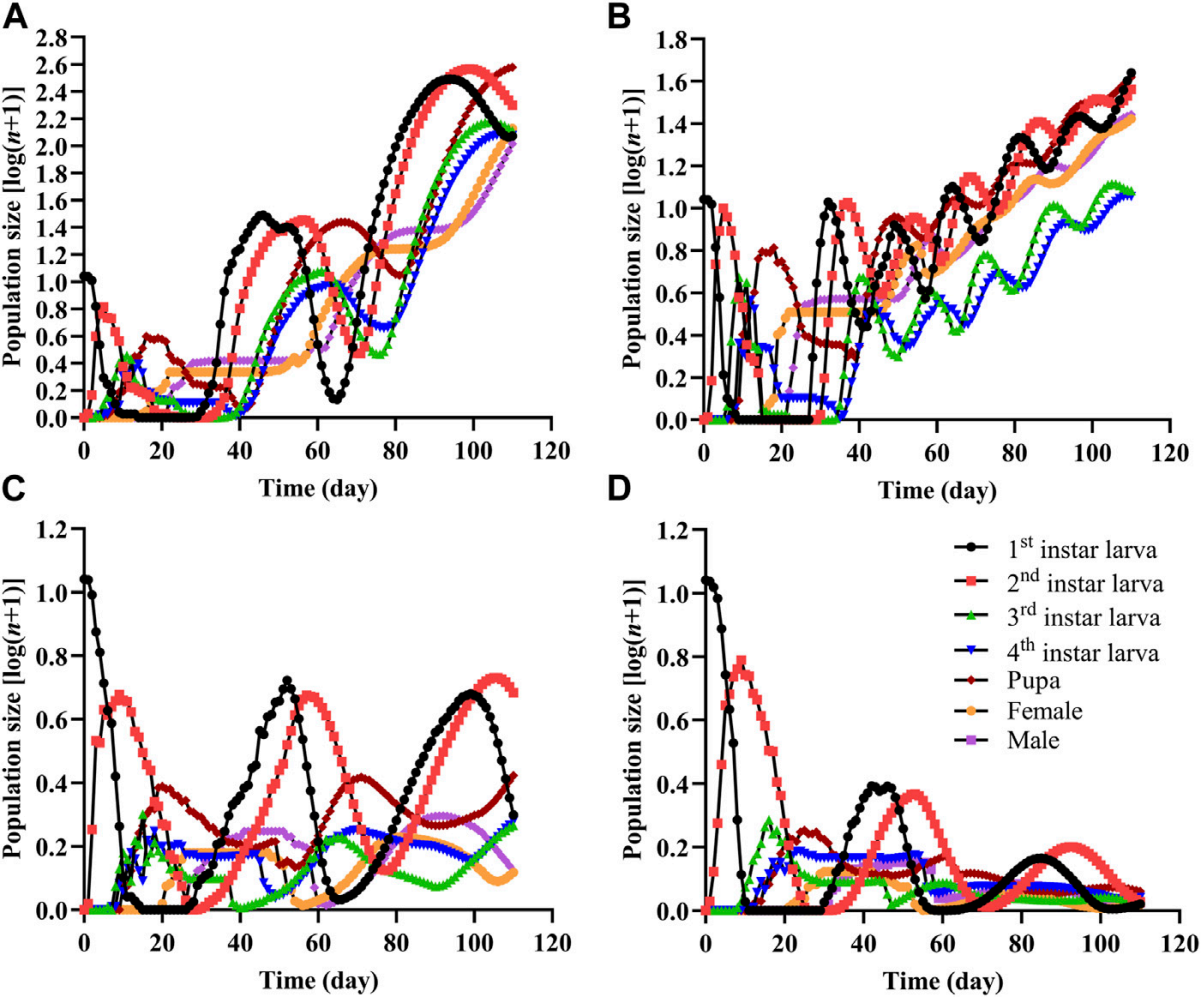
Population projection of Colorado potato beetle under water stress. An initial population of 10 eggs was used in each projection. **(A)** CK, **(B)** LD, **(C)** MD, and **(D)** HD.

The population projection showed the stage sizes and the emergence of different stages (Figure 4). The growth of the Colorado potato beetle population was faster and the population size was larger on CK and LD than on the MD and HD treatments. The total adult population sizes on CK and LD were 235.4 and 52.2 individuals, respectively, while the populations barely increased on MD (1.6 individuals) and HD (0.7 individuals).

## 4 Discussion

The effects on insects after host plant water stress may be either positive or negative (Dai, 2016). A large number of studies have shown that water stress is not conducive to insect oviposition. Salgado and Saastamorinen (2019) found that *Melitaea cinxia* L. under water stress before the *Plantago lanceolata* L. spawning preference is weak. This study found a similar conclusion that water stress was not favorable for Colorado potato beetle laying eggs, with adults laying more eggs on the potato plants suitable for water treatment and fewer eggs on the water-treated potato plants. Awmack and Leather. (2002) found that insect selection for their host plants includes adult oviposition selection and larval feeding selection. Generally, adults will choose host plants that are more suitable for the growth and development of their offspring to lay eggs. The oviposition selection of Colorado potato beetles adults is beneficial to the survival and development of larvae; that is, the growth and development of Colorado potato beetles larvae are better when they feed on potato plants with appropriate water treatment and LD. Hahn and Maron. (2018) found that feeding on water-stressed plants was beneficial to the growth and development of *Danaus plexippus* L. larvae. However, Showler and Moran (2003) found that *Spodoptera exigua* Hübner larvae had poorer growth and higher mortality after feeding on cotton treated with water stress. Valim et al. (2016) fed drought-stressed cabbage to *Plutella xylostella* L. and found that larval survival, pupa weight, reproductive rate, and population growth rate were reduced. In this experiment, LD had a positive effect on the growth and development of Colorado potato beetle, and feeding on leaves under LD was beneficial to the survival and pupation of Colorado potato beetle. Moderate and heavy drought negatively affected the growth and development of Colorado potato beetle, and feeding on leaves treated with water stress was not beneficial for survival and pupation of Colorado potato beetle, and the number of adults was low. Studies have found that the water content in plant leaves has a direct impact on the feeding and growth of plant-feeding insects, and water stress reduces the water content of plant leaves. If plants are in a state of water shortage for a long time, insects will have not only a longer development period after feeding but also a lower survival rate (Connor, 1988; Lower and Orians, 2003; Bisigato et al., 2015), and the results of this study are similar to those of previous studies.

This study also found the reduced Colorado potato beetle population growth under water stress, and when Colorado potato beetles were fed on leaves after water stress treatment, the net reproductive rates, intrinsic rates of increase, and finite rates decreased significantly compared with CK, which is consistent with the previous study showing that *Sitobion avenae* Fabricius showed a lower net reproductive rates, intrinsic rates of increase, and finite rates of increase on plants grown under water stress (Xie et al., 2020). Demographic parameters can be used to predict population growth: the intrinsic rate of increase reflects the growth ability of the population and can be used to measure the trend of population growth and decline at that time or in the future (Ahn et al., 2020; Liao et al., 2022a). In this experiment, the intrinsic rate of increase of Colorado potato beetles under CK treatment of 0.0436 was significantly higher than that of LD, MD, and HD treatments, indicating that water stress was not conducive to the growth of the Colorado potato beetle population.

This paper analyzed the oviposition selection and growth development of Colorado potato beetles reared in groups after water stress treatment of the host plant potato. It showed that moderate and heavy drought had adverse effects on Colorado potato beetles, delaying and reducing the population growth of Colorado potato beetles. However, this study only discussed the effects of water stress of host plants on the egg laying selection, growth, development, and reproduction of Colorado potato beetles. However, in actual production, temperature and natural enemies can affect the population growth of Colorado potato beetles. Therefore, the effects of water stress on the population growth and survival of natural enemies of Colorado potato beetles under experimental conditions need further study.

## 5 Conclusion

Water stress affects the oviposition behavior, growth and development, survival, and reproduction of insects. In this study, it was found that water stress significantly affected the developmental duration, reproduction, and the population growth of Colorado potato beetles, and they laid more eggs on potato plants suitable for water treatment. The development period of Colorado potato beetles was prolonged with the increase in water stress, and the survival rate and the amount of eggs were reduced. The intrinsic rates of increase (*r*), finite rates of increase (*λ*), and net reproductive rates (*R*_0_) of the Colorado potato beetles decreased. There was no significant difference between the LD and the control. The MD and severe drought had adverse effects on the growth and reproduction of the Colorado potato beetles. Adult egg laying preference in Colorado potato beetles is consistent with offspring performance, so the relationship between oviposition preference and offspring performance in Colorado potato beetles under water stress supports the “preference–performance hypothesis.”

## Data Availability

The original contributions presented in the study are included in the article/Supplementary Material; further inquiries can be directed to the corresponding author.
